# Association Between Housing Status and COVID-19 Severity, Morbidity, and Mortality in the United States: A Propensity Score-Matched Analysis of the National Inpatient Sample

**DOI:** 10.7759/cureus.80731

**Published:** 2025-03-17

**Authors:** Alexandra Millhuff, Mohammed A Quazi, Abdullah W Mamdani, Mahnoor Waqar, Rimmel Ali, Rayika Syed, Adeel Nasrullah, Abu Baker Sheikh

**Affiliations:** 1 Internal Medicine, University of New Mexico School of Medicine, Albuquerque, USA; 2 Psychiatry and Behavioral Sciences, University of New Mexico, Albuquerque, USA; 3 School of Medicine, University of New Mexico, Albuquerque, USA; 4 Internal Medicine, Rawalpindi Medical University, Rawalpindi, PAK; 5 School of Medicine, Dow Medical College, Karachi, PAK; 6 Pulmonary and Critical Care Medicine, Allegheny General Hospital, Pittsburgh, USA

**Keywords:** complications, covid-19, homeless, housing status, morbidity, mortality, national inpatient sample, unstable housing

## Abstract

The influence of unstable housing on COVID-19 health outcomes remains inadequately elucidated in existing literature. Prevailing assumptions suggest that individuals with unstable housing are more likely to have limited access to healthcare services, exacerbated comorbid conditions, and suboptimal living environments. Utilizing the 2020 National Inpatient Sample, we conducted a retrospective analysis on a cohort of 3838305 patients diagnosed with COVID-19. Of these, 22550 (0.59%) were identified as residing under unstable housing conditions. Both multivariate regression and propensity score-matched analyses were employed to evaluate key outcomes: in-hospital mortality, complications, hospitalization charges, length of stay, and disposition statuses. Contrary to the extant literature on infectious diseases, our analysis unveiled that patients with unstable housing conditions had lower rates of in-hospital mortality and fewer complications compared to those with stable housing. Notwithstanding, the propensity of this subgroup to leave against medical advice poses unique challenges and introduces the potential for adverse outcomes. While the findings contribute nuanced perspectives, it is imperative to recognize that unstable housing continues to be a significant determinant of a constellation of long-term health issues, such as mental health disorders, substance use, and chronic illnesses. Consequently, targeted interventions are indispensable for enhancing the health trajectory of this vulnerable population, especially in the exigencies of the COVID-19 pandemic.

## Introduction

As of August 16th, 2023, there have been 769806103 cases globally of coronavirus disease or COVID-19, including 6955497 deaths, reported by the World Health Organization [1]. With millions of confirmed cases worldwide, a comprehensive understanding of the risk and protective factors for COVID-19 is helpful for the prevention of disease infection, progression, and adverse outcomes [2]. Some potential factors identified include male sex, lower socioeconomic status, ethnicity, smoking, and certain comorbidities such as hypertension, cardiovascular disease, chronic lung disease, diabetes, and obesity [3]. Given the infectious disease risks for people experiencing unstable housing, it is critical to understand the true burden of COVID-19 in this population in order to inform prevention recommendations and provision of care [4].

Homelessness affects more than 580000 Americans on any given night, and people experiencing unstable housing are more likely to have multiple chronic medical or mental health conditions [5]. The US Interagency Council on Homelessness estimated approximately 15000 COVID-19 cases and 250 deaths among individuals with unstable housing in 2020 [6]. Risks of transmission in unstable housing communities as well as barriers to preventive behaviors such as social distancing and regular handwashing may place these individuals under higher vulnerability to COVID-19 infection [7].

The aim of our study is to determine the effects of COVID-19 on unstable housing using the National Inpatient Sample (NIS) Healthcare Cost Utilization Project (HCUP), which is supported by the Agency for Healthcare Research and Quality (AHRQ). The NIS is the largest publicly available inpatient US healthcare database and accounts for approximately 20% of all US hospital discharges. The dataset includes weights for producing national and regional estimates [8]. Improved comprehension of the relationship between housing status and COVID-19 is indispensable for developing targeted interventions to mitigate the healthcare burden confronting this vulnerable demographic.

## Materials and methods

In this retrospective study, we analyzed the NIS data that covers hospitalizations between 1 January 2020 to 31 December 2021. This data became publicly available in October 2023. By accessing this database, we obtained de-identified billing and diagnostic codes from participating hospitals. It is important to note that the NIS dataset is compliant with federal regulations and guidelines, as it does not involve the direct involvement of human subjects [9]. Consequently, it is exempt from requiring approval from an institutional review board.

The NIS database documented approximately 32.3 million discharges during this timeframe, of which 1.67 million were patients admitted due to COVID-19 within the United States. Following the exclusion of cases with missing data for key variables, our retrospective study encompassed a sample of 3838305 patients. Of these, 3815755 (99.4%) resided in stable housing conditions, while 22550 (0.6%) were characterized by unstable housing. In this study, we use a combined definition encompassing both "homelessness" and "inadequate housing stability." Homelessness refers to the condition where individuals lack a fixed, regular, and adequate nighttime residence. Inadequate housing stability denotes situations where individuals or families have housing, but it fails to meet basic standards of quality, safety, or health, such as residences with structural defects, missing essential services (e.g., heating, electricity, or running water), or conditions presenting health risks (e.g., mold and pests). The combined definition signifies continuous or recurrent risks of displacement or a lack of permanency arising from either state, due to economic challenges, eviction threats, or other external factors. This integrated approach draws from ICD codes Z59.0 (Homelessness), Z59.1-3, and Z59.8 (Inadequate housing) and aligns with a broader conceptual understanding of housing instability.

To examine associations between the two patient cohorts and categorical variables, we employed the chi-square test for independence. Simple linear regression models were used for continuous response variables, while logistic regression models were employed for binary response variables, retaining independent variables with p-values equal to or less than 0.2 in both cases. Given the imbalanced sample sizes between the two cohorts under study, propensity score matching (PSM) was implemented. Subsequently, the analyses were reiterated employing identical regression models and statistical tests. Propensity matching was conducted in R, using baseline demographics and Elixhauser comorbidity indices as matching criteria. For the purposes of data analysis and statistical modeling, we utilized the Python and R programming languages, augmented by SAS (formerly Statistical Analysis System) software. In the final models, p-values less than 0.05 were deemed statistically significant.

## Results

Demographics and baseline characteristics

In our comprehensive study including 3838305 hospitalized COVID-19 patients, we identified a subset of 22550 patients (0.59%) living under unstable housing circumstances. Most of the subjects were stable housing patients, accounting for 99.41% (3815755) of the total hospitalizations.

Notably, the unstable housing group demonstrated an overrepresentation of males, accounting for 72.42% (1961195) compared to 51.4% (1854560) in the stable housing group (p < 0.001). This group was also significantly composed of African American (27.29% (n = 6155) vs. 17.30% (n = 660060), p < 0.001) and Native American (2.31% (n = 520) vs. 0.81% (n = 0940), p < 0.001) individuals. We observed a predominance of patients in the 50-69 years (45.96% (n = 10365) vs. 38.23% (n =1458670), p < 0.001), 30-49 years (33.68% (n=7595) vs. 18.76% (n = 715950), p < 0.001), and 18-29 years (8.8% (n = 1985) vs. 5.44% (n = 207540), p < 0.001) age groups within the cohort experiencing unstable housing. The mean age for females and males in this group were 50.68 ± 17.9 years and 52.06 ± 14.6 years, respectively, and were significantly linked to lower median household incomes below $51999 (43.66% (n = 9845) vs. 33.15% (n = 1265080), p < 0.001).

Those affected by both COVID-19 and unstable housing exhibited significantly higher rates of smoking (59.76% (n = 13475) vs. 27.12% (n = 1034805), p < 0.001), alcohol use (23.68% (n=5340) vs. 2.32% (n= 88575), p < 0.001), and drug misuse (32.0% (n = 7215) vs. 2.18% (n = 83075), p < 0.001). Among their comorbidities profile, this population also reported a higher prevalence of chronic pulmonary disease (25.21% (n = 5685) vs. 21.53% (n = 821560), p < 0.001) and depression (17.07% (n = 3850) vs. 10.67% (n = 407070), p < 0.001) compared to patients in stable housing who presented with a higher incidence of obesity (28.76% (n = 1097460) vs. 14.75% (n = 3325), p < 0.001), hypertension (61.71% (n = 2354865) vs. 47.34% (n = 10675), p < 0.001), diabetes (37.38% (n = 1426400) vs. 24.66% (n = 5560), p < 0.001), and coronary artery disease (CAD) (16.78% vs. 11.31%, p < 0.001). The unstable housing population was covered by Medicaid (46.21% (n = 640260) vs. 14.61% (n = 2550), p < 0.001) and more frequently presented in urban teaching hospitals (84.83% (n = 19130) vs. 69.27% (n = 2643170), p < 0.001). A detailed summary of the baseline demographics for the cohorts studied can be found in Table 1.

**Table 1:** Baseline characteristics of COVID-19-positive housed and unhoused cohorts

Characteristics	COVID-19 and housed		COVID-19 and unhoused		P-value
	N	%	N	%	
N = 3838305	3815755	99.41	22550	0.59	
Gender (%)					
Female	1854560	48.6	6220	27.58	<0.001
Male	1961195	51.4	16330	72.42	<0.001
Mean age years (SD)	Mean	SD	Mean	SD	<0.001
Female	61.54	18.82	50.68	17.89	<0.001
Male	62.08	16.55	52.06	14.64	<0.001
Age groups (%)					
18-29	207540	5.44	1985	8.8	<0.001
30-49	715950	18.76	7595	33.68	<0.001
50-69	1458670	38.23	10365	45.96	<0.001
≥70	1433595	37.57	2605	11.55	<0.001
Race (%)					
Asian or Pacific Islander	111725	2.93	510	2.26	<0.001
Black	660060	17.30	6155	27.29	<0.001
Hispanic	688895	18.05	2955	13.10	<0.001
Native American	30940	0.81	520	2.31	<0.001
Other	137625	3.61	930	4.12	<0.001
White	2186510	57.30	11480	50.91	
Median household income (%)					
≤51,999	1265080	33.15	9845	43.66	<0.001
52K-65999	1036545	27.16	5510	24.43	<0.001
66K-87999	880370	23.07	4325	19.18	<0.001
≥88k	633760	16.61	2870	12.73	<0.001
Insurance status (%)					
Medicaid	557300	14.61	10420	46.21	<0.001
Medicare	1826210	47.86	6400	28.38	<0.001
No charge	9665	0.25	180	0.8	<0.001
Other	167255	4.38	1175	5.21	<0.001
Private Insurance	1108415	29.05	1985	8.8	<0.001
Self-pay	146910	3.85	2390	10.6	<0.001
Hospital division (%)					
East North Central	579640	15.19	2495	11.06	<0.001
East South Central	268015	7.02	1105	4.9	<0.001
Middle Atlantic	527255	13.82	4035	17.89	<0.001
Mountain	247825	6.49	2080	9.22	<0.001
New England	134745	3.53	1535	6.81	<0.001
Pacific	442600	11.6	1795	7.96	<0.001
South Atlantic	858870	22.51	5285	23.44	<0.001
West North Central	216540	5.67	1915	8.49	<0.001
West South Central	540265	14.16	2305	10.22	<0.001
Hospital bedsize (%)					
Large	1722665	45.15	11930	52.9	<0.001
Medium	1119315	29.33	5820	25.81	<0.001
Small	973775	25.52	4800	21.29	<0.001
Hospital teaching status (%)					
Rural	409955	10.74	865	3.84	<0.001
Urban nonteaching	762630	19.99	2555	11.33	<0.001
Urban teaching	2643170	69.27	19130	84.83	<0.001
Comorbidities (%)					
Coronary artery disease	640260	16.78	2550	11.31	<0.001
Myocardial infarction	156915	4.11	895	3.97	0.279
Essential hypertension	2354865	61.71	10675	47.34	<0.001
Diabetes	1426400	37.38	5560	24.66	<0.001
Cancer	166735	4.37	515	2.28	<0.001
Obesity	1097460	28.76	3325	14.75	<0.001
Drug abuse	83075	2.18	7215	32	<0.001
Smoking	1034805	27.12	13475	59.76	<0.001
Alcohol	88575	2.32	5340	23.68	<0.001
Chronic pulmonary disease	821560	21.53	5685	25.21	<0.001
Peripheral vascular disease	155080	4.06	610	2.71	<0.001
Chronic kidney disease	526025	13.79	1670	7.41	<0.001
Hypothyroidism	486975	12.76	1335	5.92	<0.001
Autoimmune	123330	3.23	390	1.73	<0.001
Depression	407070	10.67	3850	17.07	<0.001
AIDS	17085	0.45	455	2.02	<0.001
Dementia	348040	9.12	1225	5.43	<0.001

We applied PSM, considering variables like patient age, gender, race, income, insurance status, and Elixhauser comorbidities. After this process, each group (those with stable housing and those experiencing unstable housing conditions) was comprised of 22550 patients.

In-hospital mortality

In our multivariate logistic regression model, salient variables such as age, hospital bed size, race, gender, hospital location, hospital teaching status, hospital regional positioning, median household income, primary expected payer, and Elixhauser comorbidities were accounted for, as outlined in Table 2. Interestingly, our data revealed significantly lower in-hospital mortality among COVID-19 patients with unstable housing compared to stable-housed COVID-19 patients (4.15% (n = 935) vs. 13.07% (n = 498900), adjusted OR (aOR): 0.39 (95% CI 0.34-0.45), p < 0.001). These findings persisted after the application of PSM.

**Table 2:** In-hospital outcomes for COVID-19-positive housed and unhoused cohorts *Adjusted for age, hospital bed size, race, gender, hospital location, hospital teaching status, hospital region, median household income, expected primary payer (insurance status), and Elixhauser comorbidities. AKI, acute kidney injury; VTE, venous thromboembolism; CVA, cerebrovascular accident

Characteristics	COVID-19 and housed		COVID-19 and unhoused		Adjusted odds ratio	95% CI	P-value
	N	%	N	%	NA	NA	NA
N = 3838305	3815755	99.41	22550	0.59	NA	NA	NA
Disposition (%)	N	%	N	%	NA	NA	NA
Against medical advice	52965	1.39	1870	8.29	NA	NA	<0.001
Discharged alive unknown destination	1710	0.04	5	0.02	NA	NA	<0.001
Home health care	525655	13.78	705	3.13	NA	NA	<0.001
Routine	2014060	52.78	13715	60.82	NA	NA	<0.001
Transfer other	618025	16.2	4455	19.76	NA	NA	<0.001
Transfer to short-term hospital	104440	2.74	865	3.84	NA	NA	<0.001
Complications (%)	N	%	N	%	NA	NA	NA
Acute liver failure	42860	1.12	125	0.55	0.35	0.24-0.52	<0.001
Sudden cardiac arrest	104855	2.75	245	1.09	0.41	0.31-0.52	<0.001
Mean total hospitalization charge ($)	$98930.51	NA	$71491.14	NA	Adjusted total charge*=$32699.02 lower for unhoused	NA	<0.001
Mean length of stay (days)	8.25	NA	9.4	NA	Adjusted length of stay*=0.70 days higher for unhoused	NA	<0.001
In-hospital mortality (n=210,640)	498900	13.07	935	4.15	0.39	0.34-0.45	<0.001
Vasopressor use	99870	2.62	260	1.15	0.38	0.29-0.50	<0.001
AKI	1062390	27.84	4290	19.02	0.63	0.58-0.68	<0.001
VTE	227210	5.95	1030	4.57	0.73	0.63-0.84	0.254
Hemodialysis	173390	4.54	505	2.24	0.48	0.39-0.58	<0.001
Invasive mechanical ventilation	476120	12.48	1395	6.19	0.41	0.36-0.46	<0.001
Non-invasive mechanical ventilation	271625	7.12	560	2.48	0.44	0.36-0.53	<0.001
CVA	68650	1.80	225	1.00	0.45	0.34-0.61	<0.001
Tracheostomy	51140	1.34	75	0.33	0.18	0.11-0.30	<0.001
AMI	75490	1.98	225	1.00	0.56	0.41-0.75	0.037

In-hospital complications

A comprehensive evaluation of several in-hospital complications was carried out. The cohort with COVID-19 and unstable housing showed a significantly lower incidence of acute kidney injury (AKI) (19.0% (n = 4290) vs. 27.84% (n = 1062390), aOR: 0.64 (95% CI 0.58-0.68), p < 0.001), reduced rate of vasopressor use (1.15% (n = 260) vs. 2.62% (n = 99870), aOR: 0.38 (95% CI 0.29-0.5), p < 0.001), and fewer instances of hemodialysis (2.24% (n = 505) vs. 4.54% (n = 173390), aOR: 0.48 (95% CI 0.39-0.58), p < 0.001). Similarly, the occurrences of invasive (6.19% (n = 1395) vs. 12.48% (n = 476120), aOR: 0.41 [95% CI 0.36-0.46], p < 0.001) and non-invasive mechanical ventilation (2.48% (n= 560) vs. 7.12% (n = 271625), aOR: 0.44 (95% CI 0.36-0.53), p < 0.001) were less frequent within the unstable housing group. The same group also reported lower rates of cerebrovascular accident (CVA) (1.0% (n = 225) vs. 1.8% (n = 68650), aOR: 0.45 (95% CI 0.34-0.61), p = 0.001) and AMI (1.0% (n = 225) vs. 2.0% (n = 75490), aOR: 0.56 (95% CI 0.41-0.75), p = 0.001). The incidence of venous thromboembolism (VTE) was also lower in the unhoused groups (4.57% (n = 1030) vs. 5.95% (n = 227210), aOR: 0.73 (95% CI 0.63-0.84), p = 0.001). After PSM, these findings persisted (Table 3).

**Table 3:** In-hospital outcomes of a propensity-matched sample *Adjusted for age, hospital bed size, race, gender, hospital location, hospital teaching status, hospital region, median household income, expected primary payer (insurance status), and Elixhauser comorbidities.

Characteristics	COVID-19 and housed		COVID-19 and unhoused		Adjusted odds ratio	95% CI	P-value
	N	%	N	%	NA	NA	NA
N = 45100	22550	50	22550	50	NA	NA	NA
Disposition (%)	N	%	N	%	NA	NA	NA
Against medical advice	1160	5.14	1870	8.29	NA	NA	<0.001
Discharged alive unknown destination	10	0.04	5	0.02	NA	NA	<0.001
Home health care	2155	9.56	705	3.13	NA	NA	<0.001
Routine	12995	57.63	13715	60.82	NA	NA	<0.001
Transfer other	3380	14.99	4455	19.76	NA	NA	<0.001
Transfer to short-term hospital	780	3.46	865	3.84	NA	NA	<0.001
Complications (%)	N	%	N	%	NA	NA	NA
Acute liver failure	310	1.37	125	0.55	0.037	0.23-0.59	0.001
Sudden cardiac arrest	560	2.48	245	1.09	0.44	0.31-0.62	<0.001
Mean total hospitalization charge ($)	$96655.12	NA	$71491.14	NA	Adjusted total charge* = $30005.80 lower for unhoused	NA	<0.001
Mean length of stay (days)	8.25	NA	9.4	NA	Adjusted length of stay* = 0.57 days higher for unhoused	NA	<0.001
In-hospital mortality (n = 1265)	2070	9.18	935	4.15	0.40	0.33-0.48	<0.001
Vasopressor use	675	2.99	260	1.15	0.35	0.26-0.49	<0.001
AKI	5745	25.48	4290	19.02	0.64	0.58-0.72	<0.001
VTE	1185	5.25	1030	4.57	0.82	0.67-1.00	0.045
Hemodialysis	990	4.39	505	2.24	0.47	0.36-0.60	<0.001
Invasive mechanical ventilation	3115	13.81	1395	6.19	0.38	0.33-0.45	<0.001
Non-invasive mechanical ventilation	1075	4.77	560	2.48	0.50	0.40-0.63	<0.001
CVA	490	2.17	225	1	0.45	0.32-0.64	<0.001
Tracheostomy	315	1.4	75	0.33	0.20	0.12-0.36	<0.001
AMI	430	1.91	225	1	0.48	0.33-0.70	<0.001

In-hospital quality measures and disposition

The mean length of hospital stay was marginally longer for the cohort experiencing unstable housing (9.4 days vs. 8.25 days, adjusted length of stay: 0.70 days longer for the unstable housing group, p < 0.001). The mean total charge for hospitalization was less for the unstable housing group ($71491 vs. $98,930, adjusted total charge: $32699 lower for the unstable housing group, p < 0.001).

In terms of discharge outcomes, those experiencing unstable housing had significantly higher percentages of discharges against medical advice (8.29% (n = 1870) vs. 1.4% (n = 52965), p < 0.001) and more routine discharges (60.82% (n = 13715) vs. 52.78% (n = 2014060), p < 0.001). This group had higher rates of transfers to other healthcare facilities (19.76% (n = 4455) vs. 16.2% (n = 618025), p < 0.001), lower discharges to home health care (3.13% (n=705) vs. 13.78% (n = 525655), p < 0.001), and higher admissions to short-term hospitals (3.84% (n = 865) vs. 2.74% (n = 104440), p < 0.001). Even after applying PSM, these trends persisted.

## Discussion

Our research provides an enlightening perspective that diverges from traditional associations between unstable housing and adverse health outcomes. In the mainstream discourse, individuals with unstable housing are often depicted as having reduced access to healthcare, compounded comorbidities, and living conditions not conducive to maintaining good health [10-12]. However, this literature primarily centers around chronic health conditions, overlooking the implications of acute infectious diseases like COVID-19. Contrary to this prevailing narrative, our study uncovers a rather unexpected correlation: unstable housing does not necessarily portend worse COVID-19 outcomes. Our analysis, in fact, indicates that hospitalized COVID-19 patients with unstable housing experienced lower in-hospital mortality rates and fewer complications compared to those with stable housing. This result challenges the established notion that unstable housing invariably leads to poor health outcomes and warrants deeper investigation.

Our data show lower rates of both mortality and hospital complications among COVID-19 patients from unstable housing backgrounds compared to those from stable housing, which contradicts results from two independent studies conducted in San Francisco and New York City during the COVID-19 pandemic, which reported an increase in the number of deaths among the unstable housing population [13,14]. However, a more nuanced exploration into the causes of these deaths provides some clarity. The San Francisco study attributed the rise in deaths during the pandemic primarily to overdoses, not COVID-19 [13]. The number of deaths from overdoses spiked dramatically during the pandemic, with fentanyl appearing significantly in many toxicology reports [8,13]. The New York City study also discovered that drug-related deaths outranked COVID-19 as the leading cause of death among the unstable housing population [14]. This evidence suggests that the increased rate of mortality observed among those with unstable housing in these cities was due to drug overdoses, not COVID-19. These results suggest that the unstable housing population may have been less susceptible to severe outcomes from the virus than initially assumed. Furthermore, the high mortality associated with substance abuse in these reports emphasizes an urgent need for healthcare providers and systems to address such substance use disorders.

This surprising result may be explained by the “Inoculum Theory,” which posits that the initial dose of viral exposure may impact disease severity [15,16]. Less time spent in enclosed spaces among the homeless population led to lower virus exposure, which aligns with the study conducted by Montgomery et al., which found that people experiencing homelessness (PEH) hospitalized for COVID-19 exhibited lower rates of invasive mechanical ventilation and mortality than the general population. The outdoor lifestyle of the homeless population might have led to lower virus exposure due to less time spent in enclosed spaces. This hypothesis aligns with a third study conducted by Montgomery et al, which found that PEH hospitalized for COVID-19 exhibited lower rates of invasive mechanical ventilation and mortality than the general population [12]. According to Ruiz et al, Hispanics have a mortality benefit despite preexisting health conditions due to biological, behavioral, psychological, and social factors [13]. This could also be explained by acculturation, which can serve as a proxy for social behaviors and cultural values. Further investigation is warranted among these risk factors to explain the resilience experienced by individuals in unstable housing populations.

While the authors advocate for the expansion of medical respite to reduce hospitalizations in these disproportionately affected populations, their findings also underscore the potential protective effect of homelessness against severe COVID-19 outcomes. Contrastingly, our study showed that COVID-19 patients living in unstable housing had significantly higher rates of smoking, alcohol use, and drug use. While these factors typically correlate with poorer health outcomes, they did not seem to contribute to higher mortality or hospital complications in our cohort. This unexpected outcome could potentially be due to the unique physiological responses of these individuals or the healthcare system’s effective management of these patients. However, the salient finding from the New York and San Francisco studies, which reports higher mortality from drug overdoses among the homeless population during the pandemic, again accentuates the urgency of addressing substance use disorders, particularly during periods of increased stress and isolation like the COVID-19 pandemic [14,15,17]. The decreased use of substance use disorder services during the pandemic, as reported by Scarlett and colleagues and in the San Francisco study, further supports this recommendation [14]. Targeted interventions such as mobile health clinics, hospital discharge planning that is specific for unhoused patients, and support with coordination and transportation for medical follow-up appointments may help address this substance abuse crisis.

Nonetheless, the actual incidence of mortality and complications among those in unstable housing situations could surpass our study’s reported rates due to their increased tendency to leave against medical advice or endure disruptions in care. This propensity might result in under-detection or under-reporting, which could contribute to the ostensibly lower in-hospital mortality and complication rates that we observed. The repercussions of these circumstances can be grave, leading to potentially adverse health outcomes such as hospital readmission or even death. Prior research confirms that patients from unstable housing are more prone to leaving against medical advice (AMA), thereby heightening their likelihood of encountering adverse health outcomes [18,19]. A confluence of factors, such as the absence of social support, financial instability, and mental health challenges, is likely to underpin this trend. The issue of patients leaving AMA is particularly pressing among those with unstable housing and necessitates decisive intervention [19]. Providing increased social support, financial assistance, and mental health treatment could be key to addressing this issue. Aiding these deficits may improve health outcomes for this vulnerable demographic, affirming our commitment to holistic patient care and equity in healthcare provision.

While our study has been able to illuminate several facets of housing instability and its relationship with COVID-19 outcomes, there are limitations to be aware of. Primarily, our utilization of the 2020 NIS data set brings inherent constraints. The NIS offers a sizable and diverse patient pool, yet only provides insight into inpatient experiences. Therefore, we were unable to account for COVID-19 patients who received outpatient care or were not hospitalized. Our study, being retrospective and observational in nature, limits the extent to which causative relationships can be established. Furthermore, the potential exists for inaccuracies within the NIS coding system. Our ability to correctly identify patients with unstable housing and their related health outcomes hinged on the precise use of ICD-10 codes, the reliability of which can fluctuate. Our identification of unstable housing situations may also lack full breadth due to the confines of the ICD-10 coding system, as there is potential for overlap, where individuals might fit into both categories, or may overlook certain unstable housing conditions such as "couch surfing" leading to ambiguities. Moreover, this data relies on self-reporting, which can be influenced by stigma and reluctance to disclose, potentially leading to an underrepresentation or misclassification of this demographic and, consequently, affecting our conclusions. Despite these limitations, a strength of our study is that it captures a large sample size across different regions in the US, warranting the ability to examine national trends and specific populations such as patients with unstable housing. Furthermore, the use of PSM in our statistical analysis can help mitigate confounding variables between housed and unhoused patients. Despite this, there may have been residual confounding variables in our study due to unmeasured factors such as nutritional status, social support, or access to healthcare prior to hospitalization. 

No consistent or standardized method for screening patients for housing stability exists [20,21]. Studies have shown that ICD-10 codes may be more specific than sensitive indicators of housing status, inconsistently map housing status screening instruments, and that Z-codes specific to housing status are found in only 1-2% of inpatient medical records [22-26]. Additionally, reliance on ICD-10 codes for defining housing status introduces a possible misclassification bias, as housing status may not be accurately documented. Despite these limitations, this prior work supports the results of this study as being the “tip of the iceberg” for which a better data method for housing status does not exist. Consistent with previous work [27,28], ICD-10 codes remain an important metric for measuring health disparities and are being used ubiquitously across US hospitals [29].

Additionally, the data set, while expansive, lacked granularity in certain potential influencers such as primary healthcare access, nutritional status, social support networks, and other social determinants of health. Finally, our study was unable to track post-discharge outcomes like mortality, complications, or long-term effects of COVID-19.

Moving forward, we advocate for subsequent studies to employ prospective designs and more comprehensive data collection methodologies to validate our findings and delve deeper into the complex relationship between COVID-19 outcomes and housing instability. As our research heavily relied on ICD-10 codes, future research efforts could benefit from refining methods for identifying and characterizing unstable housing situations. Such enhancements can not only increase research precision but also help shape more effective interventions tailored to this demographic. Our study’s findings also urge the incorporation of a wider range of social determinants of health in future research to better understand their influence on health outcomes. Long-term follow-up studies that monitor patients beyond their hospitalization can provide essential insights into the enduring health impacts of COVID-19 among individuals with unstable housing. This information could then be used to strategize management and prevention of potential long-term effects of COVID-19. Finally, given the geographical diversity within the United States, it is vital for future studies to explore how local healthcare practices and public health policies impact health outcomes among the population with unstable housing. Such research could aid in designing regional-specific interventions to mitigate the health impacts of housing instability in the context of COVID-19.

## Conclusions

Our study highlights the complex interplay between housing instability, demographics, and health outcomes, particularly for COVID-19 patients. These findings underscore the importance of further research to gain a thorough understanding of the role of housing instability in health outcomes and to develop effective, targeted strategies to improve the health of this vulnerable population, especially in the context of the COVID-19 pandemic.
